# Stress-related white matter microstructure alterations and chronic pain

**DOI:** 10.1007/s11682-026-01102-4

**Published:** 2026-02-07

**Authors:** Yann Quidé, Khaizuran Kamarul, Sylvia M. Gustin

**Affiliations:** 1 NeuroRecovery Research Hub, School of Psychology, UNSW Sydney, Sydney, NSW 2052 Australia; 2 Centre for Pain IMPACT, Neuroscience Research Australia, NSW 2031 Randwick, Australia

**Keywords:** Trauma, Stress, Diffusion, Chronic pain

## Abstract

Posttraumatic stress symptoms (PTSS) are commonly experienced in people with chronic pain. Reduced white matter microstructural integrity in the uncinate fasciculus and the cingulum has separately been reported in chronic pain and posttraumatic stress disorder (PTSD) studies. However, the relationship between chronic pain, PTSS and white matter integrity remains unclear. This study aims to disentangle the relationship between PTSS severity and white matter microstructural integrity common across different chronic pain conditions. Thirty-six subjects with chronic pain and 20 without chronic pain (controls) underwent diffusion weighted imaging and completed the civilian version of the PTSD CheckList (PCL-C). Average fractional anisotropy (FA) values were extracted from the uncinate fasciculus, and the cingulate and hippocampal portions of the cingulum. A series of multiple linear regressions determined the main effects of group, PTSS severity (PCL-C total score) and their interactions on each region separately. The group-by-PTSS interaction was significantly associated with uncinate fasciculus FA variations. Moderation analysis indicated that increasing PTSS severity was significantly associated with reduced uncinate fasciculus FA in the control group, but not in the chronic pain group. No other significant association was found for any other ROI FA values. Consistent with previous studies, increasing PTSS levels were associated with reduced FA of the uncinate fasciculus in controls, but not in people with chronic pain. Other mechanisms may be at play in chronic pain, including the interplay with other psychopathological problems or specific pain type effects.

## Introduction

Chronic pain has severe impacts on mental health and quality of life (Cohen et al., 2021). Around 20% of people with chronic pain experience posttraumatic stress symptoms (PTSS), and chronic pain is experienced by 20% to 80% of people with posttraumatic stress disorder (PTSD) (Brennstuhl et al., 2015; Siqveland et al., 2017). Chronic pain and chronic stress, including PTSD/PTSS, are associated with overlapping morphological brain alterations, including variations in grey matter volume in the hippocampus, amygdala, medial prefrontal cortex (mPFC), the middle cingulate cortex (MCC) and the posterior insula (Abdallah & Geha, 2017; Quidé et al., 2025).

The medial temporal lobe is physically connected to the mPFC via two distinct pathways. The cingulum consists of two main tracts, the *hippocampal* portion (CGH) connecting the hippocampus to the posterior cingulate cortex (PCC), and the *cingulate* portion of the cingulum (CGC), connecting the PCC to the mPFC via the MCC and the dorsal anterior cingulate cortex (Catani et al., 2013). On the other hand, the uncinate fasciculus is directly connecting the amygdala and the mPFC (Catani et al., 2013).

Common alterations in white matter fractional anisotropy (FA), a marker of microstructural integrity, have been reported across various chronic pain conditions (Bautin et al., 2025). Chronic neuropathic pain following spinal cord injury is associated with reduced cingulum and uncinate fasciculus FA (Guo et al., 2019; Ilvesmäki et al., 2017), whereas lower cingulum FA is evident in trigeminal neuralgia (DeSouza et al., 2014). Similarly, lower FA in both the cingulum and the uncinate fasciculus was observed in temporomandibular disorder (Budd et al., 2022). However, other studies have reported no differences in white matter microstructure integrity in any of these tracts (Gustin et al., 2010; Lieberman et al., 2014; Parise et al., 2014; Tan et al., 2019), suggesting that chronic pain alone may not be the only factor contributing to these alterations in white matter integrity.

Reduced FA in the same white matter tracts has been reported in PTSD or in association with PTSS (Daniels et al., 2013; Dennis et al., 2021; Siehl et al., 2018). In studies of trauma-exposed individuals, combat-exposed veterans who did not develop PTSD showed decreased FA in the uncinate fasciculus compared to controls (McCunn et al., 2021), whereas severity of PTSS following medical trauma was associated with lower uncinate fasciculus (one-month post-trauma) and cingulum FA (three months post-trauma) (Harnett et al., 2020). Finally, decreased posterior cingulum FA was observed in African American women with PTSD (Fani et al., 2012). However, another study reported no association between white matter integrity changes and PTSS (Weis et al., 2021).

The present study sets out to characterise the relationship between PTSS severity and white matter microstructural integrity common across different types of chronic pain conditions, and how this relationship differs compared to people who do not experience chronic pain. We hypothesised that the relationship between FA values in the cingulum (CGC, CGH) and uncinate fasciculus, and variations in PTSS would be different in people with chronic pain compared to controls without chronic pain; in other words, we expected that group (controls versus chronic pain) would moderate the relationship between variations in PTSS and variations in FA in the selected ROIs. We expected that the cumulative effect of chronic pain and PTSS would be evident in lower FA values in these tracts for people with chronic pain compared to control.

## Methods

### Power analysis

Power analysis using G*Power v3.1.9.6 (Faul et al., 2009; Faul et al., 2007) indicated that a minimum of 54 participants was necessary (F(5,48) = 3.09, λ = 18.90), and thus confirmed that our sample (*n* = 56) was of sufficient size to achieve 80% power for detecting a large (f^2^ = 0.35) effect for 5 predictors and accounting for the number of white matter regions studied (α = 0.05/3 = 0.017).

### Participants

In this cross-sectional, convenience sample, participants were 36 people with chronic pain conditions lasting for more than three months (together referred to as the *chronic pain* group), including temporomandibular disorder (TMD; *n* = 14), trigeminal neuropathic pain (TNP; *n* = 8), burning mouth (*n* = 1), trigeminal neuralgia (*n* = 6), temporomandibular disorder + trigeminal neuropathic pain (*n* = 1), and spinal cord injury neuropathic pain (*n* = 6), as well as 20 people who never experienced chronic pain (controls, HC). Neuropathic pain after spinal cord injury was diagnosed according to the International Association for the Study of Pain Spinal Cord Injury Pain Taxonomy (Bryce et al., 2012). All people with spinal cord injury suffered from a complete paraplegia with continuous burning and/or shooting pain in areas of sensorimotor loss. Painful TMD is characterised by ongoing musculoskeletal facial pain as assessed using the Research Diagnostic Criteria for TMD (Dworkin & LeResche, 1992). TNP and postherpetic neuralgia, both characterised by continuous dull neuropathic facial pain with sharp exacerbations, were diagnosed using the Liverpool Criteria (Nurmikko & Eldridge, 2001).

Inclusion criteria for all participants were age over 18 years old with no known diagnosis of psychiatric disorder. Exclusion criteria included heart pacemaker, metal implants, intrauterine contraceptive device, insulin pump, infusion devices, hearing-disease, claustrophobia, pregnancy, a history of stroke, multiple sclerosis, or Parkinson’s disease; and a history of chronic pain for HCs. All participants were volunteers who provided informed consent according to procedures approved by the Human Research Ethics committees of the University of New South Wales (HC15206), the University of Sydney (HREC06287) and Northern Sydney Local Health District (1102-066 M).

### Assessments

The PTSD Checklist – Civilian (PCL-C) (Weathers et al., 1993) is a standardized self-report 17-item questionnaire with good psychometric properties in non-clinical (psychiatric) populations (Conybeare et al., 2012), used to indicate how much participants have been bothered by stressful life experiences over the past month using a 5-point scale (1 = not at all, 5 = extremely). In this study, no provision of a formal PTSD diagnosis was intended, and criterion A was not assessed; participants were not explicitly asked about their experience of specific traumatic events. The PCL was used to capture symptom severity rather than to diagnose PTSD, and interpretation will be made in the context of stressful, rather than posttraumatic stress, symptoms. First, the total score to the PCL-C (PCL-C total score, ranging from 17 to 85) was used, and then associations with the severity of specific symptoms were explored: re-experiencing (cluster B; questions 1–5, ranging from 5 to 25), avoidance (cluster C; questions 6–12, ranging from 7 to 35) and hyperarousal symptoms (cluster D; questions 13–17, ranging from 5 to 25). The use of these specific PTSS scores was intended to describe the severity of specific PTSS. Severity of depressive symptoms was measured using the Beck Depression Inventory (BDI-I total score; ranging from 0 to 63) (Beck et al., 1961), and the severity of state anxiety was assessed using the State Anxiety Inventory (SAI total score; ranging from 20 to 80) (Spielberger et al., 1983).

Participant’s pain intensity was measured using a visual analogue scale (VAS) in two ways. First, a ‘pain diary’ index averaging experienced levels of pain on a 10-cm horizontal ruler (‘no pain’ = 0; ‘worst pain imaginable’ = 10) three times a day (morning, noon, and evening) for seven days prior to their scanning session. Second, a ‘scan pain’ index was used to retrospectively measure pain intensity during scanning (asked immediately after leaving the scanning room).

### Magnetic resonance imaging

Imaging data were collected with two Phillips 3 T Achieva TX scanner (Philips Healthcare, Best, The Netherlands) housed at Neuroscience Research Australia (Randwick, NSW, Australia) or at St Vincent’s Hospital (Darlinghurst, NSW, Australia), both equipped with 8-channel head coils. At both sites, a 3D T1-weighted MPRAGE structural scan covering the entire brain (acquisition parameters: repetition time (TR) = 5.6 ms, echo time (TE) = 2.5 ms, field of view (FOV) = 250 × 250 × 174 mm, 288 × 288 matrix, 200 sagittal slices, flip angle = 8°, voxel size = 0.9 × 0.9 × 0.9 mm) and diffusion weighted imaging (DWI) were acquired (acquisition parameters: spin echo-planar imaging (EPI) acquisition sequence, 32 directions, b = 1000 s/mm2, TE/TR = 87/8879.79 ms, FOV = 224 × 137.5 × 224 mm, resolution = 2 mm isotropic).

Diffusion images were pre-processed following standard protocols from the Enhancing NeuroImaging Genetics through Meta-Analysis (ENIGMA) consortium (https://github.com/ENIGMA-git), using tools from the Functional MRI of the Brain Software Library (FSL; version 6.0.5.2) (Jenkinson et al., 2012).

Data were first denoised using the Marchenko-Pastur principal component analysis (MP-PCA) (Veraart et al., 2016) and corrected for Gibbs ringing (Kellner et al., 2016) using tools from the MRtrix3 software (respectively, *dwidenoise* and *mrdegibbs*) (Tournier et al., 2019). Because only one phase encoding was collected, Synb0-DisCo (Schilling et al., 2019) was used to create a synthetic b0 map from the T1-weighted image. Eddy currents were corrected using FSL *eddy*, and tensors were fitted with FSL *dtifit*. After each pre-processing step, the quality of images was assessed to identify potential errors in image processing using the ENIGMA guidelines. Finally, diffusion metrics such as fractional anisotropy (FA) and mean diffusivity (MD) were generated using FSL *tract-based spatial statistics* (TBSS) following the ENIGMA guidelines.

Average FA values for bilateral uncinate fasciculus, bilateral cingulate (CGC) and hippocampal (CGH) portions of the cingulum, were extracted from the John Hopkins University (JHU) white matter atlas. Before statistical analyses, extracted FA values were harmonized for scanner differences using the python-based neuroHarmonize tools (https://github.com/rpomponio/neuroHarmonize) (Pomponio et al., 2020), controlling for age, sex, group, and PCL-C total score.

### Statistical analyses

A series of multiple linear regressions was performed using the *lm_robust* function from the ‘estimatr’ R package (v 1.0.4) (Blair et al., 2024) to determine the main effects of group (HC versus chronic pain), of the severity of stressful symptoms (PCL-C total score) and their interaction (the product of group-by-mean-centred PCL-C total score), on harmonized FA values of each ROI (one model per ROI). Age and sex were added as covariates in all analyses. Only models surviving Bonferroni correction to account for the number of ROIs (uncinate fasciculus, CGC, CGH) were considered (*p* = 0.05/3 = 0.017). All analyses were performed in R (v4.3.1) (R Core Team, 2023) and RStudio (2023.6.2.561) (Posit Team, 2023).

In case of significant associations between the group-by-stress interaction and FA values, moderation analyses (group as moderator) were performed using the ‘interactions’ R package (v1.1.5) (Long, 2021). The Davidson–McKinnon correction (HC3) was used to account for heteroskedasticity (Hayes & Cai, 2007) using the R package ‘sandwich’ (v3.2.2) (Zeileis, 2004; Zeileis et al., 2020). Within each significant model, statistical significance was set at a threshold of *p* < 0.05.

### Exploratory analyses

Symptom-specific effects were also explored using scores for the re-experiencing, avoidance and hyperarousal scales. Additional Bonferroni correction was applied to the original corrected threshold for significance to account for the number of symptoms studied for each ROIs (*p* = 0.017/3 = 6 × 10^−3^). 

## Results

### Participant characteristics

Demographic details are summarised in Table 1. Briefly, participants with chronic pain were not statistically different from the HC group in terms of age, sex and scanning site distributions. However, they reported more severe stressful, depression, and anxiety symptoms than the HC group. We note that all analyses were conducted using complete-case data only; participants with missing values for variables relevant to a given analysis were excluded.

**Table 1:** Sociodemographic and clinical characteristics of the studied cohort

	HC (*N* = 20)	Chronic Pain (*N* = 36)	Statistics Welch/t/χ^2^	df	*p*-values		
Age, in years, mean (SD) [range]	50.48 (15.17) [24.2–80.86]	52.17 (13.30) [23.77–74.81]	−0.431	54	0.668		
Sex, n (F/M)	14/6	28/8	0.415	1	0.536		
PCL-C Total score (SD) [range]	21.95 (5.10) [17–40]	31.36 (9.63) [17–52]	**−4.780**	**53.94**	**< 0.001**		
PCL-C Re-experiencing score (SD) [range]	6.15 (1.63) [5–11]	7.81 (3.37) [5–17]	**−2.472**	**53.29**	**0.008**		
PCL-C Avoidance score (SD) [range]	8.80 (2.82) [7–19]	13.06 (4.50) [7–28]	**−4.344**	**53.08**	**< 0.001**		
PCL-C Hyper-arousal score (SD) [range]	7.00 (1.59) [5–10]	10.50 (4.00) [5–19]	**−4.630**	**50.23**	**< 0.001**		
BDI total score (SD) [range]^#^	3.61 (3.09) [0–10]	10.40 (7.18) [0–29]	**−4.743**	**48.58**	**< 0.001**		
STAI-State, mean (SD) [range]^$^	29.63 (6.80) [22–45]	35.09 (8.69) [22–57]	**−2.351**	**50**	**0.011**		
Pain condition (TMD/TNP/BM/TRIG/TMD + TNP/SCI)	-	14/8/1/6/1/6	-	-	-		
Pain duration, in years, mean (SD) [range]^	-	10.03 (9.73) [1–36]	-	-	-		
VAS pain diary, mean (SD) [range] ^%^	-	3.98 (2.21) [0.2–8.4]	-	-	-		
VAS scan pain, mean (SD) [range]^%^	-	3.56 (2.29) [0–9]	-	-	-		
Scanning sites (NeuRA/SVH)	14/6	29/6	1.233	1	0.319		
**FA values for each ROI**							
UNC, mean (SD) [range]	0.534 (0.044) [0.458–0.651]	0.521 (0.061) [0.314–0.661]	0.877	54	0.384		
CGC, mean (SD) [range]	0.607 (0.031) [0.549–0.647]	0.616 (0.035) [0.519–0.679]	−0.986	54	0.328		
CGH, mean (SD) [range]	0.543 (0.032) [0.501–0.618]	0.540 (0.047) [0.418–0.620]	0.347	51.743	0.730		

*HC* healthy controls, *Pain* individuals with chronic pain, *df* degrees of freedom, *SD* standard deviation, *F/M* females/males, *BDI* Beck Depression Inventory, *STAI* State-Trait Anxiety Inventory, *TMD/TNP/BM/TRIG/TMD + TNP/SCI* temporomandibular disorder/trigeminal neuropathic pain/burning mouth/trigeminal neuralgia/temporomandibular disorder+trigeminal neuropathic pain/spinal cord injury, *VAS* visual analogue scale, *NeuRA* Neuroscience Research Australia, *SVH* St Vincent’s Hospital Sydney, *FA* fractional anisotropy, *ROI* region of interest, *UNC* uncinate fasciculus, *CGC* cingulum – cingulum bundle, *CGH* cingulum – hippocampal bundle

^#^ Data missing for 2 HC and 2 chronic pain participants; ^$^ Data missing for 1 HC and 3 chronic pain participants; ^%^: Data missing for 1 chronic pain participants; ^ missing data for 4 chronic pain participants

Significant group differences are in bold

### Analyses for PCL-C total score

**Fig. 1:**
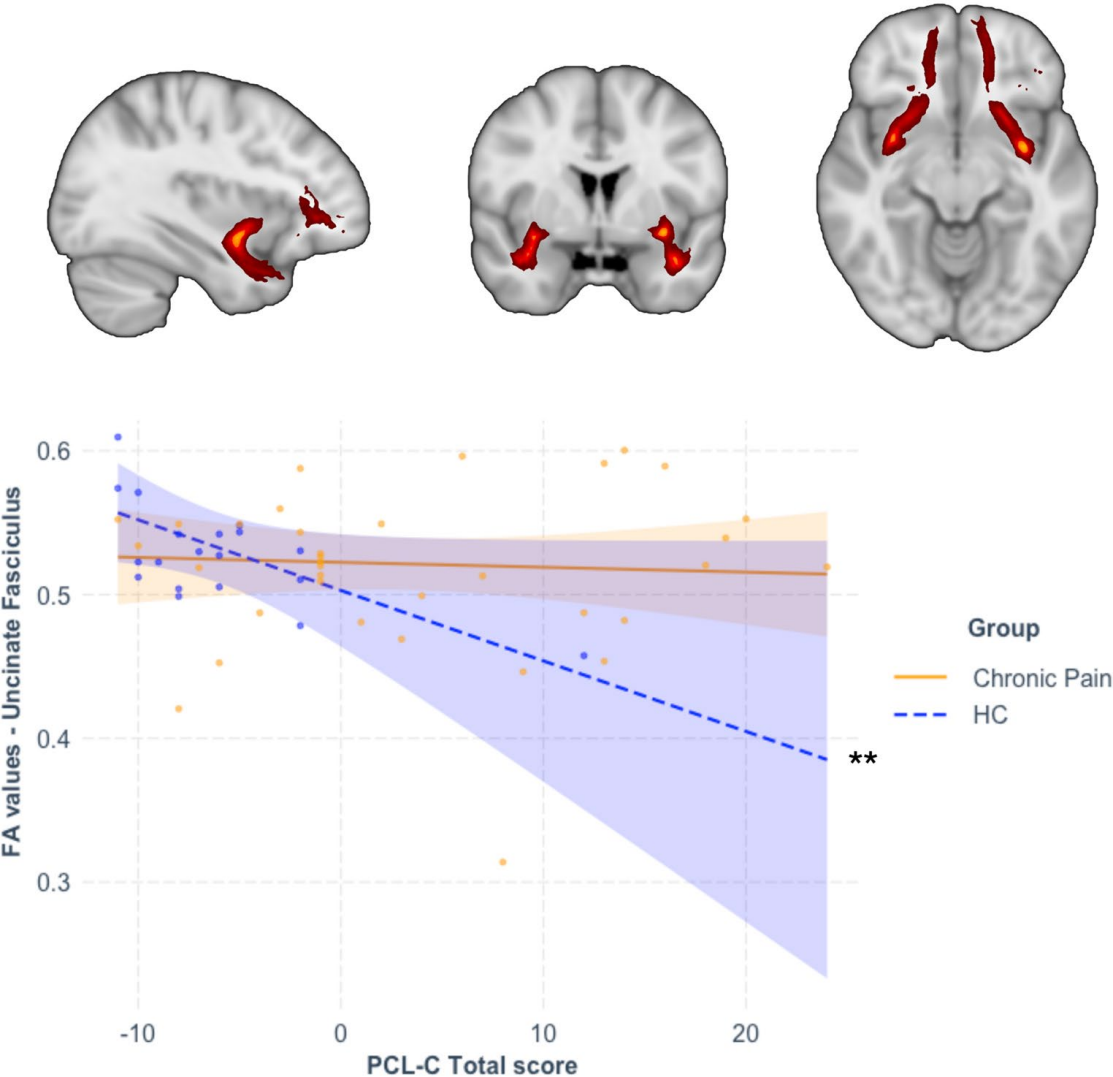
Moderation analysis following significant association between the group-by-PTSS interaction term and variations in fractional anisotropy values in the uncinate fasciculus. The interaction term was significantly associated with variations in fractional anisotropy (FA) in the uncinate fasciculus (coloured in red for illustrative purpose). Increasing severity of PTSS was significantly associated with decreased FA values in the uncinate fasciculus in the healthy control (HC group, blue dashed line), while this association was not significant in people with chronic pain (yellow plain line) ** *p* < 0.01 Coloured band around each line represents 95% confidence intervals

Table 2 summarises the results of all statistical models tested. Models for the uncinate fasciculus and the CGC were significant, but only the model for the uncinate fasciculus survived Bonferroni correction (*p* < 0.017). Within this model, and in the context of no significant direct effects of group, the direct effect of stress severity and the group-by-stress interaction were significantly associated with variations of uncinate fasciculus FA. Moderation analysis indicated that increasing stress severity was significantly associated with decreased uncinate fasciculus FA in the HC group (*b*=−0.005, *p* = 0.001) but was not associated with variations in FA in the chronic pain group (*b*=−3 × 10-4, *p* = 0.741; see Fig. 1).

**Table 2:** Results of the moderation analyses for all ROIs

ROI	Model				Group						PTSS (PCL-C score)						Group x Trauma						
	Adj *R*^2^	F	df	*p*-value	b	se	LLCI	ULCI	t-value	*p*-value	b	se	LLCI	ULCI	t-value	*p*-value	b	se	LLCI	ULCI	t-value	*p*-value	
**PCL-C Total**																							
UNC	**0.039**	**3.521**	**5**,**50**	**0.008**	0.020	0.013	−0.006	0.045	1.521	0.135	**−0.005**	**0.001**	**−0.008**	**−0.002**	**−3.718**	**0.001**	**0.005**	**0.002**	**0.001**	**0.008**	**2.754**	**0.008**	
																**HC**	**−0.005**	**0.001**	**−0.008**	**−0.002**	**−3.718**	**0.001**	
															Chronic pain		−3 × 10-4	0.001	−0.002	0.002	−0.333	0.741	
CGC	0.138	2.687	5,50	**0.032**	0.014	0.012	−0.009	0.038	1.231	0.224	−0.001	0.001	−0.003	0.001	−0.851	0.399	0.002	0.001	−0.001	0.005	1.560	0.125	
CGH	0.040	0.595	5,50	0.704	0.008	0.017	−0.026	0.042	0.448	0.656	−0.001	0.002	−0.005	0.003	−0.646	0.522	3 × 10-5	0.002	−0.004	0.004	0.134	0.894	
**PCL-C Re-experiencing**																							
UNC	0.004	1.106	5,50	0.369	0.001	0.016	−0.030	0.032	0.063	0.950	−0.012	0.006	−0.024	0.001	−1.850	0.070	0.012	0.007	−0.002	0.025	1.681	0.099	
CGC	0.115	2.419	5,50	**0.049**	0.015	0.011	−0.007	0.038	1.358	0.181	−0.004	0.005	−0.014	0.006	−0.883	0.382	0.007	0.005	−0.004	0.017	1.255	0.215	
CGH	0.031	0.711	5,50	0.618	0.005	0.012	−0.019	0.029	0.392	0.697	−0.006	0.005	−0.016	0.004	−1.201	0.236	0.003	0.006	−0.008	0.014	0.566	0.574	
**PCL-C Avoidance**																							
UNC	0.067	1.271	5,50	0.291	0.019	0.017	−0.016	0.053	1.084	0.284	**−0.010**	**0.004**	**−0.019**	**−0.001**	**−2.148**	**0.037**	0.007	0.005	−0.002	0.017	1.531	0.132	
CGC	0.132	2.927	5,50	**0.022**	0.012	0.011	−0.010	0.035	1.129	0.264	−0.002	0.002	−0.006	0.003	−0.636	0.528	0.004	0.003	−0.001	0.009	1.430	0.156	
CGH	0.030	0.748	5,50	0.592	0.007	0.017	−0.027	0.041	0.403	0.689	−0.002	0.004	−0.010	0.006	−0.574	0.569	−2 × 10-4	0.004	−0.009	0.008	−0.038	0.970	
**PCL-C Hyperarousal**																							
UNC	0.011	0.982	5,50	0.438	0.008	0.022	−0.036	0.052	0.356	0.723	−0.009	0.007	−0.024	0.005	−1.288	0.204	0.010	0.0082	−0.005	0.025	1.328	0.190	
CGC	0.099	2.102	5,50	0.081	0.012	0.016	−0.021	0.045	0.719	0.475	−0.001	0.006	−0.012	0.010	−0.169	0.866	0.003	0.006	−0.009	0.015	0.464	0.645	
CGG	0.079	0.286	5,50	0.919	−0.003	0.016	−0.036	0.030	−0.161	0.873	1 × 10-4	0.005	−0.010	0.010	0.019	0.985	-−8 × 10-4	0.005	−0.012	0.010	−0.146	0.885	

*FA* fractional anisotropy, *ROI* region of interest, *PTSS* posttraumatic stress symptoms, *PCLC* posttraumatic stress disorder checklist - civilian, *UNC* uncinate fasciculus, *CGC* cingulum – cingulum bundle, *CGH* cingulum – hippocampal bundle, *Adj R*^2^ adjusted coefficient of determination, *se* standard error, *LLCI* bootstrapped 95% lower levels confidence interval, *ULCI* bootstrapped 95% upper levels confidence interval

Statistically significant associations (*p* < 0.05 within each model) are in bold; models surviving Bonferroni correction are highlighted in grey

### Analyses for specific stress symptoms

As shown in Table 2, the models investigating the moderating effect of group on the relationship between re-experiencing and avoidance symptoms and FA values in the CGC failed to survive Bonferroni correction (*p*<6 × 10^− 3^). No other models were significant.

## Discussion

The present study examined the differential relationship between comorbid stressful symptoms and variations in white matter microstructural integrity in the cingulum and uncinate fasciculus, in people with and without chronic pain. Relative to the control group, the chronic pain group reported overall more severe stress symptoms. In addition, the group-by-stress interaction was significantly associated with variations in uncinate fasciculus FA. More severe stress was associated with lower uncinate fasciculus FA, only in the HC group. This was in the context of a direct association between stress severity and lower FA values in the uncinate fasciculus (independently of group). There was no significant association between FA values in any of the cingulum portions (cingulate or hippocampal) and group, stress severity or their interaction.

Contrary to our hypotheses, increased severity of stress was associated with decreased microstructural integrity of the uncinate fasciculus in the control group, but not in the chronic pain group. Reduced FA in the uncinate fasciculus of our control group is in line with previous studies showing reduced FA values in this tract in people exposed to trauma who did not develop PTSD, compared to controls never exposed to trauma (O’Doherty et al., 2018). This indicates that exposure to trauma or a stressful event, even when no psychopathology develops, can impact the integrity of the uncinate fasciculus that may reflect long term resilience to stressful events. However, the lack of group difference or association between stress and FA values in the uncinate fasciculus in the chronic pain group was unexpected, suggesting that other mechanisms may be at play in chronic pain. It is possible that experiencing chronic pain alters stress processing or dampens variability in coping responses, leading to aberrant associations between stress and brain integrity. While reduced coping capacity typically increases vulnerability to stress, which might be expected to strengthen stress–brain associations, chronic pain may instead lead to neurobiological changes that influence this relationship. Lower uncinate fasciculus FA is inconsistently associated with chronic pain (Budd et al., 2022; Mišić et al., 2024), but is reported in other conditions comorbid to chronic pain, such as depression and anxiety, that may also influence the integrity of the uncinate fasciculus in chronic pain. For instance, depression impacts uncinate fasciculus FA (Xu et al., 2023), and affects the functional connectivity between the mPFC and the amygdala (Quidé et al., 2023), two regions physically linked by the uncinate fasciculus, in chronic pain. Investigation of the possible cumulative effects of different psychopathological features (depression, anxiety, stress) on brain integrity was prevented due to the relatively small sample size of this study and is warranted in future studies of larger samples.

Also surprising, was the lack of group differences and association with stress severity on cingulum integrity. It is possible that the lack of group difference in the cingulum may reflect the heterogeneity of the studied sample (Bautin et al., 2025). Although we hypothesised that group differences would be evident across pain conditions, different types (e.g., neuropathic versus musculoskeletal pain), or location of pain (e.g., localised versus widespread) may have different effects on white matter integrity. Studies of larger sample, such as through the ENIGMA-Chronic Pain consortium (Quidé et al., 2024), will help better identify the roles of these different factors. In addition, unlike for changes in grey matter integrity (Quidé et al., 2025), the role of stress severity on white matter integrity in the cingulum may also depend on the timing of trauma exposure. A recent study showed that while decreased integrity of the uncinate fasciculus is associated with stress severity a month after trauma, decreased integrity of the cingulum was evident later, three months post-trauma (Harnett et al., 2020). It is therefore possible that in absence of clinical levels of stress, variations in white matter may only be transient and normalise over time. This interpretation remains speculative, and longitudinal studies with both short (months) and long-term (years) outcomes are needed to confirm this.

This study has several limitations. First, while the sample size was large enough to test our initial hypotheses, it may also have limited our abilities to identify subtle effects and to investigate the roles of other psychopathological factors on white matter microstructural integrity. The effects of stress may be mediated by the severity of depressive or anxiety symptoms in this population. However, the sample size of this study prevented the inclusion in the same models, of depressive or anxiety symptoms, pain intensity or subtypes, without risking increasing type II errors. Second, pharmacological treatments were not accounted for in the present analyses. This is partly due to the presence of the control group in all analyses and to the limited sample size. However, potential neurotrophic or neuroprotective effects of pharmacological treatments on brain morphology, function and neurochemistry (Hunsberger et al., 2009) cannot be ruled out and must be acknowledged. Third, although it can also be considered a strength, another potential limitation is the inclusion of people with different types (e.g., neuropathic, musculoskeletal) and location of pain (e.g., lower body, head) together. This approach is the most appropriate to identify changes in white matter microstructural integrity across pain conditions, but also increases the heterogeneity of the sample and may have contributed to confounding effects pertaining to specific pain types or locations. Fourth, no measure of pain intensity was recorded for the control group. This prevented the investigation of the relationship between pain intensity and stress severity on brain integrity. Future studies should record this variable to better characterise this relationship. Finally, in the context of co-occurrent chronic pain and stress symptoms, participants may answer PCL-C questions as being about their pain, likely inflating stress ratings (e.g. Ravn et al., 2019).

## Conclusions

Contrary to our hypotheses, the severity of stress symptoms was associated with reduced white matter microstructural integrity (FA) in the uncinate fasciculus in the control group only. These findings indicate that other factors may be at play when investigating the effects of stress on brain integrity of people with chronic pain. These include changes in neuroendocrine or immune functions, medication effects and the impacts of comorbid conditions to chronic pain, such as depression or anxiety that may confound the impacts of stress on brain integrity. Disruption of the fronto-limbic circuits involved in stress regulation may have behavioural and clinical implications including alterations of top-down processes that can lead to difficulties in emotion regulations and other affective and cognitive problems associated with chronic pain. Larger studies are needed to better understand the cumulative interplay between comorbid psychopathologies and brain integrity in people with chronic pain.

## Data Availability

Although the raw data cannot be accessed due to ethical restrictions, the dataset produced in the present study can be made available upon reasonable request to the authors.
